# Omega−3 Polyunsaturated Fatty Acids (PUFAs): Emerging Plant and Microbial Sources, Oxidative Stability, Bioavailability, and Health Benefits—A Review

**DOI:** 10.3390/antiox10101627

**Published:** 2021-10-15

**Authors:** Ramesh Kumar Saini, Parchuri Prasad, Reddampalli Venkataramareddy Sreedhar, Kamatham Akhilender Naidu, Xiaomin Shang, Young-Soo Keum

**Affiliations:** 1 Department of Crop Science, Konkuk University, Seoul 05029, Korea; 2 Institute of Biological Chemistry, Washington State University, Pullman, WA 99164, USA; prasad.parchuri@wsu.edu; 3 Plant Cell Biotechnology Department, CSIR-Central Food Technological Research Institute (CSIR-CFTRI), Mysuru 570020, India; rvs@cftri.res.in; 4 Department of Biochemistry, CSIR-Central Food Technological Research Institute (CSIR-CFTRI), Mysuru 570020, India; akhilnaidu@gmail.com; 5 Jilin Provincial Key Laboratory of Nutrition and Functional Food, Jilin University, Changchun 130062, China; xmshang@jlu.edu.cn

**Keywords:** eicosapentaenoic acid (EPA), docosahexaenoic acid (DHA), chia (*Salvia hispanica*), *Echium plantagineum*, *Buglossoides arvensis*, microalgae, thraustochytrids, *Schizochytrium* sp.

## Abstract

The omega−3 (n−3) polyunsaturated fatty acids (PUFAs) eicosapentaenoic acid (EPA) and docosahexaenoic (DHA) acid are well known to protect against numerous metabolic disorders. In view of the alarming increase in the incidence of chronic diseases, consumer interest and demand are rapidly increasing for natural dietary sources of n−3 PUFAs. Among the plant sources, seed oils from chia (*Salvia hispanica*), flax (*Linum usitatissimum*), and garden cress (*Lepidium sativum*) are now widely considered to increase α-linolenic acid (ALA) in the diet. Moreover, seed oil of *Echium plantagineum*, *Buglossoides arvensis*, and *Ribes* sp. are widely explored as a source of stearidonic acid (SDA), a more effective source than is ALA for increasing the EPA and DHA status in the body. Further, the oil from microalgae and thraustochytrids can also directly supply EPA and DHA. Thus, these microbial sources are currently used for the commercial production of vegan EPA and DHA. Considering the nutritional and commercial importance of n−3 PUFAs, this review critically discusses the nutritional aspects of commercially exploited sources of n−3 PUFAs from plants, microalgae, macroalgae, and thraustochytrids. Moreover, we discuss issues related to oxidative stability and bioavailability of n−3 PUFAs and future prospects in these areas.

## 1. Introduction

Naturally occurring fatty acids (FAs) can be classified according to their carbon-chain length and the number of double bonds. Long-chain (LC) FAs contain more than 12 carbon atoms, and FAs containing 22 or more carbon atoms are sometimes referred to as very long-chain (VLC) FAs [1]. Based on the number of double bonds, FAs can be classified into saturated FAs (SFAs, no double bonds), monounsaturated FAs (MUFAs, a single double bond), and polyunsaturated FAs (PUFAs, ≥2 double bonds). In the diet, palmitic acid (C16:0) and stearic acid (C18:0) are the SFAs, and oleic acid (C18:1) is the main MUFA. The PUFAs can be further classified into two groups, omega−3 (ω−3 or n−3) and omega-6 (ω−6 or n−6), based on the position of the first double bond on the methyl terminal end [2]. For instance, α-linolenic acid (ALA, C18:3 cis-9,12,15), with the first double bond at the third position from the methyl terminal end, and linoleic acid (LA, C18:2 cis-9,12), with the first double bond at the sixth position from the methyl terminal end, are termed n−3 and n−6 FA, respectively. These FAs are essential, because they cannot be produced within the human body. In the body, LC-PUFAs (LA and ALA) are converted to VLC-PUFAs by Δ6- (FADS2) and Δ5-desaturases (FADS2), and their respective elongases (ELOVL). Stearidonic acid (SDA; C18:4 cis-6, 9, 12, 15), docosapentaenoic (DPA; C22:5 cis-7,10,13,16,19), eicosapentaenoic (EPA, 20:5 cis-5,8,11,14,17), and docosahexaenoic (DHA; C22:6 cis-4,7,10,13,16,19), are the n−3 LC- and VLC-PUFAs produced from ALA in the body [2].

Both n−3 and n−6 PUFAs play a vital role in body homeostasis. The lipid mediators derived from n−3 and n−6 PUFAs, however, may have contrasting effects on body homeostasis. In general, the higher levels of n−6 PUFAs may be associated with constriction of blood vessels, inflammation, and platelet aggregation, whereas n−3 PUFAs may help to resolve inflammation and alter the function of vascular biomarkers. However, studies have also shown that increasing n−6 PUFA intake (e.g., LA) while keeping n−3 PUFA intake unchanged has no harmful effects on either oxidative stress or markers of inflammation [3]. In an actual health scenario, having less EPA and DHA in the body is more harmful than is having too much arachidonic acid (ARA; C20:4 cis-5,8,11,14, n−6) [4]. Thus, an integrated approach should be considered for increasing the n-3 PUFAs EPA and DHA in the body.

Vegetarians who do not eat any animal meat constitute a significant minority of the world’s population [5]. Lacto-ovo-vegetarians eat eggs and/or dairy products, whereas vegans do not eat any foods derived from animals, including eggs, milk, and honey [5]. Studies have suggested that a well-planned vegan or vegetarian diet can supply all the essential nutrients for good health [6]. However, there are concerns about the risk of low intakes of some nutrients, including calcium, vitamin D, vitamin B12, and n−3 PUFAs (especially EPA and DHA) in inadequately planned and/or unfortified vegan or vegetarian diets [5,7,8]. 

The significant health benefits of EPA and DHA have led to increased demand for dietary supplements. The fatty fishes (e.g., salmon, mullet, and mackerel) are the typical source of EPA and DHA in the diet. This substantially increased the demand for fish (expansion of the aquaculture industry) and placed immense pressure on diminishing marine species. However, the pollution in the marine environment has directed research towards the other viable alternative source of n−3 PUFAs. Moreover, fish VLC-n−3 PUFAs are not useful for vegans and vegetarians. Considering these factors, n−3-containing plant seeds, EPA- and DHA-rich thraustochytrids and microalgae, and stearidonic acid (SDA, C18:4, n−3) are currently being explored for commercial production of vegan n−3 PUFAs [9,10,11]. In this review we critically discuss the plant-based sources of ALA, SDA, EPA, and DHA, microalgae, macroalgae, and thraustochytrids, as well as the issues related to oxidative stability, bioavailability, and health beneficial effects of n−3 PUFAs. 

## 2. Are All n−3 PUFAs (ALA, SDA, EPA, and DHA) Equally Beneficial for Health?

Each class of n−3 PUFAs has distinct and potentially independent functions in the cell and cellular metabolism. In humans, the administrated ALA is mostly used in energy production, fatty-acid synthesis de novo (carbon recycling), and bioconversion to VLC-PUFAs [12]. The most crucial dietary importance of ALA and SDA results from their serving as an essential precursor of the production of VLC-PUFAs (EPA and DHA). However, they are also known to play a major role in controlling serum lipid profiles and hepatic steatosis by modifying the levels of n−6/n−3 in the liver by a mechanism different from those of EPA and DHA [13,14,15], which can be directly ingested from food or dietary supplements. Dietary ingested or de novo produced EPA and DHA are incorporated in the lipid bilayer, where they play a key specific role in the cellular functions. In the membrane lipid bilayer, EPA and DHA have distinct membrane interactions (molecular locations and orientations) and thus influence the signal transduction, fluidity, lipid oxidation, and cholesterol domain formation differently [16,17]. Moreover, the specialized pro-resolving mediators (SPMs, e.g., protectins, resolvins, and maresins) derived from EPA and DHA have some similar, but also many unique effects on cell death mechanisms and anti-inflammatory and post-inflammatory resolution [18]. 

DHA interacts with the head group region in the hydrocarbon core of the bilayer membrane, whereas EPA is specifically associated with the hydrocarbon core, thus more efficiently inhibiting the propagation of free radicals through the membrane than does DHA and other triglyceride-lowering agents (e.g., fenofibrate, niacin, and gemfibrozil), and preventing lipid (e.g., low-density lipoprotein (LDL)) oxidation [19]. In contrast, DHA-derived SPMs are critically important for neuroprotection [18,20]. Likewise, recent evidence shows that consumption of EPA alone is largely associated with a reduction in cardiovascular diseases [21,22], whereas DHA is important for neonatal brain development and mental and cognitive effects [23,24].

With the critical functions of EPA and DHA in cellular protection, several clinical and epidemiological studies have witnessed the protective role of n−3 PUFAs in chronic and metabolic disorders, including cardiovascular diseases (CVD) [25,26,27,28,29], obesity [30], bipolar disorder [31], rheumatoid arthritis [32], non-alcoholic fatty-liver disease [33], cognitive impairment [34], and type 2 diabetes [35]. 

In view of cardioprotection, most studies have supported that higher body status of n−3 FAs (especially EPA and DHA) can minimize the risk of CVD [25,26,27,28,29]. However, some studies have also reported a marginal reduction in coronary heart disease (CHD) deaths (RR 0.90, 95% CI 0.81 to 1.00; 127,378 participants) and CHD events (RR 0.91, 95% CI 0.85 to 0.97; 134,116 participants) by increased intake of EPA and DHA. Moreover, a slightly reduced risk of CVD events (RR 0.95, 95% CI 0.83 to 1.07; 19,327 participants) and arrhythmia (RR 0.73, 95% CI 0.55 to 0.97; 4912 participants) were reported with increased intake of ALA [36]. Overall, the prospective and observational studies suggest that higher dietary intakes of EPA and DHA are more beneficial in decreasing the risk of CVD and stroke than are higher intakes of ALA.

In view of the vast health benefits, n−3 PUFAs have become a key dietary supplement. However, the adverse effects need to be considered when administering n−3 PUFAs [37]. PUFAs are highly prone to free radical-induced lipid oxidative degradation, leading to the production of lipid peroxides [38], which may be harmful to health under long-term exposure [37,39]; however, detailed studies on such aspects are lacking. 

## 3. A Low Conversion Rate of ALA to EPA and DHA Is a Challenge

**Figure 1:**
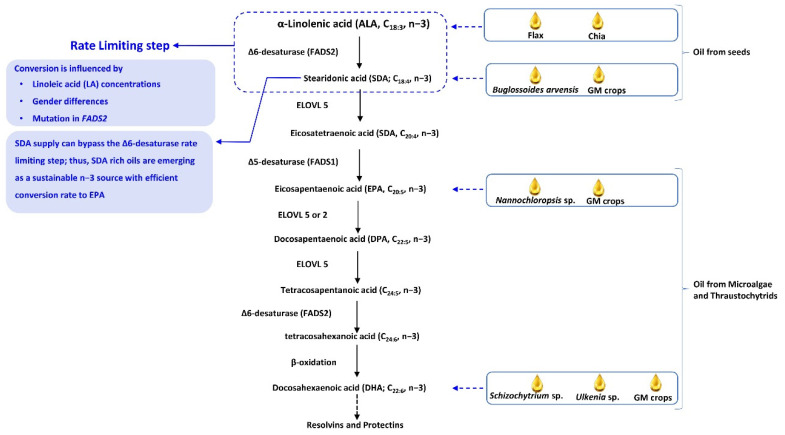
Illustrations showing that dietary supplementation of SDA (instead of ALA) can bypass the first rate-limiting step; so food rich in SDA is more beneficial than is ALA for increasing the EPA levels in the body.

In the body, the conversion of ALA to EPA and DHA is strongly limited, with a low conversion rate of 7.0–21% for EPA [40,41,42] and 0.01–1% for DHA [42,43,44]. In the body, conversion of ALA to EPA and DHA starts with Δ6-desaturase-mediated conversion of ALA to SDA. In mammals, including humans, the low bioconversion rate of ALA to EPA and DHA is largely governed by the activities of this rate-limiting enzyme Δ6-desaturase (*FADS2* gene; Figure 1). In the further steps, SDA gives rise to EPA and DHA after a series of elongase- and desaturase-mediated reactions, as shown in Figure 1. Because direct dietary supplementation of SDA (instead of ALA) can bypass the first rate-limiting step, foods rich in SDA are more beneficial than ALA is for increasing the EPA levels in the body. Cumberford and Hebard [45], mentioned that consumption of 2.3 to 3 g of SDA-rich Ahiflower oil provides the recommended minimum daily equivalents of EPA (200–250 mg), which is relatively close to that of standard marine oil (1–1.4 g) and less than that of flax or chia seed oil (5.6–11.2 g). This indicates the lower conversion rate of ALA to EPA than to SDA. Nevertheless, consuming foods rich in EPA and DHA offers advantages over ALA- and SDA-rich diets.

The highest conversion of ALA to EPA occurs when LA and ALA are supplied at the ratio of 1:1 [42]. However, in the typical western and Asian diet, this ratio is 15/1 to 16.7/1 [46]. Thus, reducing the LA intake and increasing the intake of ALA can help maintain the dietary n−6/n−3 PUFAs intake in the ratio of 1:1. In this scenario, the inclusion of ALA-rich food can substantially increase n−3 FAs in the body. Moreover, the activity of Δ6- and Δ5-desaturase enzymes is largely modulated by variants in encoding genes (FADS1-2-3 gene cluster) and determines the bioconversion rate of ALA to VLC-FUPAs [47]. For instance, the C-allele of FADS1 rs174547, related to convert plant-based LC-PUFAs into VLC-PUFAs, is dominant in Americans (59%), common in East Asians (57%) and Europeans (35%), and largely absent in South Asians (14%), and Africans (2%) [47]. These observations hypothesized that an ALA-rich diet can potentially help South Asian and African populations (regions with little access to seafood) because of the normal activities of Δ6- and Δ5-desaturase enzymes (absence of mutations) and efficient conversion of ALA to VLC-FUFAs, whereas direct supplementation of EPA and DHA may be required for the American populations, because of the low conversion rate of ALA to VLC-PUFAs [47].

## 4. The Recommended Intake of n−3 PUFAs

According to the European Food Safety Agency (EFSA) Panel on Dietetic Products, Nutrition, and Allergies (NDA), total fat intake should be within 20−35% of energy (E%) [48]. The Adequate Intake (AI) for ALA is set to 0.5 E%. Considering the health benefits of preventing death from CHD and sudden heart attacks, intake of 100 mg/d EPA plus DHA for infants (>6 months) and young children (<24 months), and 250 mg/d for adults is recommended by the EFSA Panel [48]. In addition, the panel recommended that 100–200 mg of preformed DHA should be included to provide an ample supply of n−3 VLC-PUFAs during pregnancy and lactation. The World Health Organization (WHO) also recommended 200–500 mg of EPA + DHA for adults [49], and the National Institute of Medicine (NAM; formerly known as the Institute of Medicine, IOM) suggests 10% of ALA intake should be from EPA + DHA [50]. The WHO also suggests that vegetarians who do not eat fish should ensure adequate intake of plant sources of ALA [49].

In the body, the status of n−3 FAs is measured in terms of the n−3 index, which is the red blood cell (RBC) EPA + DHA contents expressed as a % weight of total FAs [51]. An RBC n−3 index level of ≥8% is considered to be a reasonable preliminary target for reducing the risk of CHD [51]. To raise the n−3 index, increased intake of EPA and DHA-rich fish and fish-oil supplements is advised [52]. However, this option is not viable for vegans and vegetarians, for whom adding foods from plant or microbial sources can supplement the RDA of EPA and DHA.

The current evidence suggests that populations (especially vegans and vegetarians) around the world are not meeting the recommended intake of ALA, EPA, and DHA [7,8,50,53]. The National Health and Nutrition Examination Survey (NHANES; the United States, 2003–2014) data on n−3 fatty-acid intake demonstrated a substantial difference in EPA and DHA intake based on gender, age, and pregnancy status. The vulnerable populations (i.e., children and women) are consuming amounts far below the RDA. For instance, children aged 6–11 years consume ~4.5–27% RDA of the EPA + DHA specified by the IOM and the WHO [50]. Recently studies showed that dietary n−3 intakes and total n−3 erythrocyte/plasma fatty acids are found to be significantly lower in vegans and vegetarians than in fish eaters and omnivores [53]. The vegan diet is deficient in EPA and DHA and is about 50–60% lower than that of the omnivore group [7]. 

Genetic differences in the PUFA metabolism (especially mutations in the FADS1-2-3 gene cluster) influence the bioconversion of ALA to VLC-PUFAs [47]. Hence, these factors should be considered before setting recommendations for intake of ALA, EPA, and DHA. For mutations in the FADS1-2-3 gene cluster, which limit bioconversion of ALA to n−3, VLC-PUFAs should be considered [47].

## 5. Dietary Sources of Vegan n−3 PUFAs

### 5.1. ALA-Rich Seeds

Vegetable oil, commonly obtained from seeds (endosperm) or sometimes from fruits (e.g., palm oil and olive oil), is a significant contributor of fat in the body. Globally, per capita, 18.15 kg of vegetable oil are consumed annually [54]. Vegetable oils are dominated by sunflower, soybean, and palm oil, followed by rapeseed oil. Palm (palmolein) oil is mainly composed of oleic (43%), palmitic (40%), and LA (11%) [55]. LA (54.17%) is predominantly found in soyabean oil with the minor presence of ALA (n−6/n−3 PUFAs ratio of 10.5) [55]. In contrast, canola oil (produced from low erucic-acid (<2%) cultivars of rapeseed) is mainly composed of oleic acid (54.0–61.0%), followed by LA (20.6–25.0%) and ALA (8.7–9.5%) with an n−6/n−3 PUFAs ratio of 1.9–2.5 [56]. Like soybean and palm oil, most vegetable oils contain a significant amount of MUFAs in the form of oleic acid (especially in olive, corn, safflower, and sunflower oil) [57], which impart good thermal and oxidative stability during storage and culinary preparations. Flax (linseed; *Linum usitatissimum* L., family Linaceae) seeds containing 35–50% oil [58] are a rich source of ALA (39.0 to 60.4% of total FAs) with low contents of SFAs (9–11%) [59,60]. In addition to the oil, flax stem is a vital source of industrial high-strength fiber [58], nutritionally important tocopherols, proteins, and antioxidants [59]. As a functional food ingredient, flax or flaxseed oil is commonly incorporated into baked goods, juices, dairy products, and dry pasta products [59].

Like flax, oil obtained from chia (*Salvia hispanica*), camelina (*Camelina sativa*), and garden cress (*Lepidium sativum*) seeds also contains high proportions of ALA, thus gaining popularity as ALA-rich oil. Chia is an annual herbaceous plant that belongs to the Lamiaceae family. The Chilean chia seeds contain 30–33% oil rich in ALA (62–64% of total FAs) [61,62], whereas the Indian grown chia variety CHIAmpion-B contains 28–30% oil with ~65% ALA of total FAs [63,64]. Chia seed is also a good source of protein (15–25%), total dietary fiber (34–37%), minerals, and natural antioxidants, such as carotenoids, tocopherols, polyphenols, and phytosterols [61,65,66,67]. With the presence of a large amount of phenolic compounds, chia seed and oil exhibit good antioxidant activity [61]. Chia seed oil has GRAS status and has a good potential for supplying the demand of ALA [68]. Under the appropriate agronomic conditions, chia plants can yield 1250–1500 kg of seeds/ha [62]. Chia seeds can be considered to be a vital source of ALA for the diet. Moreover, chia seeds as a whole and flour, oil, and gel are already used in different foods, especially in baked and dairy products [69]. Owning to its gel-forming and water-absorbing properties, chia-seed gum has wide application as an emulsifier and stabilizer in the food and pharmaceutical industries [69]. 

*Camelinasativa*, also known as camelina, false flax, or gold of pleasure, is an ancient cultivated and underused Brassicaceae oilseed crop [70] with high levels of ALA (19–43% of total FAs) and low contents of SFA (5–10%) in seeds [71]. With high seed yields (up to 3300 kg/ha), camelina is also a viable source of ALA in the diet [70,71]. Moreover, camelina seed oil is conventionally used as food, feed, fuel, and in industrial applications [70]. 

Garden cress (*Lepedium sativum*) is a fast-growing edible, underused herb belonging to the Cruciferae family. Garden cress seeds contain about 21–24% oil; the oil (garden cress oil, GCO) has 32% of ALA, a balanced ratio of MUFA/PUFA (~1:1), and a good amount of natural antioxidants, such as tocopherols (1.7 mg/g) and phytosterols (12.16 mg/g). GCO is more stable than are ALA-rich flax seed, chia, and camelina oils, because of its relatively low ALA and the presence of a balanced ratio of MUFA/PUFA [72]. Blending GCO with other vegetable oils (sunflower oil, rice bran oil, sesame oil) exhibited an increase in ALA content and decrease in the LA/ALA ratio, and improved the nutritional quality of oil [73]. Dietary feeding of GCO blended oil significantly modulated fatty-acid and lipid profiles in Wistar rats. GCO and its blended oils significantly increased ALA, EPA, and DHA content in serum, liver, heart, and brain in rats [73]. ALA from GCO and its blended oils were well absorbed and metabolized to LC-PUFAs. Thus, GCO is a potential oil for ALA; it can be blended to enrich vegetable oils to obtain a desired and balanced n–6/n–3 PUFA ratio with beneficial health properties.

The oil obtained from flax, chia, camelina, and garden cress seeds is a valuable source of ALA packed with natural antioxidants, including carotenoids, tocopherols, and sterols. However, despite the presence of a good amount of natural antioxidants, oil extracted from these sources is easily oxidized when exposed to oxygen and heat, and thus cannot be used as cooking oil and in food fortification [59,74]. To overcome this problem, microencapsulation is paving a way to stabilize the ALA-rich oil in food products [74]. Microencapsulation of garden cress oil protected it against autoxidation. Biscuits supplemented with encapsulated GCO showed higher ALA content with good sensory and nutritional quality (Umesha et al. 2015).

In recent years, new sources such as tree peony (Paeonia section Moutan DC.), sacha inchi (*Plukenetia volubilis* Linneo), perilla (*Perilla frutescens*), and *Eucommia ulmoides* seeds, have been investigated for their richness in ALA. Tree peony (Paeonia section Moutan DC.) is indigenous to China and is widely grown for ornamental and medicinal purposes. Tree peony seeds are important for high oil content (27%), with more than 90% unsaturated fatty acids, especially ALA (26.1–54.7% of total FAs) [75]. Moreover, tree peony has a high seed yield of up to 491.4 g/tree [75], which makes it a potential emerging candidate for n−3 fatty acids-rich vegetable oil. 

Sacha inchi (*Plukenetia volubilis* Linneo), also known as the Inca peanut or sacha peanut, is an oleaginous perennial plant that belongs to the family Euphorbiaceae. It has been cultivated for centuries by the indigenous population of the Peruvian Amazon [76]. In recent years, sacha inchi oil has been gaining immense popularity as a rich and balanced source of n−3/n−6 PUFAs [76]. A high oil content of 33.4–37.6% has been reported in seeds of sacha inchi cultivars, mainly composed of ALA (37.3–44.2% of total FAs) and LA (35.2–41.0%) [77]. 

Perilla (*Perilla frutescens*, family Lamiaceae) is a valuable annual herb native to Southeast Asia and Indian highlands that is widely cultivated in Korea and other Asian regions for its aromatic foliage. Perilla seeds contain 30–45% oil rich in ALA (50–64%) [60]. Perilla foliage is nutritionally bioactive [78,79] and is often used as a spicy vegetable in soups, pickles, and salads, as well as for condiments and garnishes, and offers abundant pharmacological properties [79]. 

Basil (*Ocimum basilicum* L.) is mainly cultivated as an aromatic, medicinal herb in the tropical regions of Asia, Africa, and Central and South America [80,81]. Interestingly, basil seeds contain 33.0% oil, rich in ALA (57–71%) [60,80]. 

*Eucommia ulmoides* Oliver, the only living species of the genus *Eucommia* of the Eucommiaceae family, is cultivated in Japan, Korea, and China, for medicinally important foliage and bark [82]. The seeds are obtained as a byproduct of *E. ulmoides* cultivation. Interestingly, these seeds contain a high amount of oil (30–40%), rich in ALA (56–63%) and vitamin E (191 mg/100 g) [82].

English walnut (9–10% of ALA) and hemp seeds (8.8% of ALA) are also alternative sources of ALA. Although they contain a lower concentration of ALA than that of other plant-seed oils mentioned above, they can still boost overall ALA intake [8].

Currently, in view of global availability, canola, chia, flax, camelina, and garden cress seed oil can be supplemented in low-heat cooking (but are not suitable for high-heat cooking, because of low oxidative stability), spreads, and ice creams to enrich ALA content in the diet.

### 5.2. ALA-Rich Herb: Purslane 

Herbs (photosynthetic leaves) contain high proportions of ALA (>50% of total FAs); however, they are generally deficient in total lipids (2–4%, dry weight) [83,84], thus are not a significant source of ALA. Exceptionally, Purslane (*Portulaca oleracea* L., family Portulacaceae), a common weed in field crops and lawns, is a very rich source of ALA. The whole purslane plant (stems and leaves) is traditionally eaten as a green leafy vegetable in different parts of the world [85]. Among the green leafy vegetables, purslane contains the highest amount of ALA (41–66% of total FAs in leaves) with an appropriate balance with n−6 fatty-acid γ-linolenic acid (GLA) [86]. In cultivated and wild purslane genotypes, balanced n−6/n−3 ratios of 1:1–1:3 have been reported [85,87]. In addition, purslane contains a high amount of minerals, essential amino acids, and carotenoids [85]. A fast-growing weed, purslane can yield 33,000 kg/ha [87]. Thus, purslane can serve as an affordable source of ALA. However, the high content of oxalic acid and nitrates in the leaves has limited its commercial exploitation for ALA [88]. 

### 5.3. SDA-Rich Seeds and Herbs

SDA, an intermediate of ALA, is a rate-limiting step in the production of EPA and DHA in mammals. There is unusually high activity of Δ6-desaturase among the plants belonging to Onagraceae, Saxifragaceae, Scrophulariaceae, Boraginaceae, Primulaceae, and Cannabaceae families. A direct supply of SDA can bypass the Δ6-desaturase rate-limiting step and form EPA and DHA. Thus, SDA-rich oils are emerging as a sustainable source of n−3 VLC-PUFA, especially for EPA [89]. 

In the past decade, seeds of *Echium plantagineum*, *Buglossoides arvensis*, and *Ribes* spp. have been widely investigated as a source of SDA-rich oil. Purple viper’s bugloss (*Echium plantagineum*, family Boraginaceae) seeds contain 24% oil rich in ALA (34.5% of total FAs), SDA (11.0%), and GLA (9.6%), and phytosterols [90]. *E. plantagineum* oil is commercially marketed as n−3, n−6, and n−9 PUFA containing oil, a possible alternative to fish oil. In a survey of several Boraginaceae species, the highest contents of SDA were recorded in the seed oil of *Echium* (14.7%) and *Lappula patula* (13.6%) [91]. 

*Buglossoides arvensis* (L.) I.M. Johnst. (corn gromwell; Ahiflower^®^, Boraginaceae family) seeds are important by virtue of their 16–21% oil rich in SDA (17–21%) and ALA (42–50%) [45,89,92]. However, the crop yield is much less (650 to 450 kg of seeds/ha) than that of other crops [45]. The daily consumption of 11–12 g of refined Ahiflower oil per day (2.25 g SDA per day) is GRAS [45]. Interestingly, GLA dominates in some other species of the Boraginaceae family. For instance, in *Borage officinalis* L., GLA content is 15.7–34.5% of total fatty acids, and SDA and ALA accumulate as minor fatty acids (0.1–0.3 and 0.1–0.6%, respectively)[93]. *Borage officinalis* L. is considered a valuable source of GLA. 

*Ribes* spp. belonging to the Grossulariaceae family (order Saxifragales) are also a rich source of SDA [94]. Among the seeds, various cultivars of blackcurrant (*R. nigrum* L.), redcurrant (*R. rubrum* L.), gooseberry (*R. uva-crispa* L.), and jostaberry (*R. nidrigolaria* Bauer) investigated for fatty-acid composition, the highest amounts of SDA (5.6% of total fatty acids) and total n−3 PUFA (33.4%), and the lowest ratio of n−6/n−3 (1.17) PUFAs were recorded from jostaberry[94].

The foliage of in vitro grown *Mertensia maritima* (L.) Gray (family Boraginaceae) contains a significant amount of oil (10.9% DW) rich in SDA (6.0% of total lipids) and ALA (30.4%) [95]. This plant is commonly known as the oyster plant, because of the oyster-like taste of the edible foliage. The presence of high contents of SDA and GLA in oyster-plant leaves suggests that this plant can be a potential source of SDA and GLA. Among the seeds of various *Mertensia* sp. screened for the fatty-acid composition, the highest contents of SDA (9.3% of total lipids) and ALA (12.9%) were recorded in *M. alpine* (Torr.) G. Don [91].

### 5.4. Thraustochytrids, Microalgae, and Macroalgae: Source of EPA and DHA

Thraustochytrids, a heterotrophic fungus-like clade of *Stramenopiles* [96], are a commercially important source of dietary EPA and DHA. In the literature, they are referred to as algae. However, there is no phylogenetic, biological, or ecological justification for calling them ‘algae’ [96]. Thraustochytrids, especially species of genus *Schizochytriumm*, *Aurantiochytrium*, *Crypthecodinium*, and *Ulkenia*, are widely used for the commercial production of vegan EPA and DHA (Table 1) [97,98,99]. Some *Schizochytriumm* sp. can accumulate a significant amount of EPA and DHA (16.18 and 33.72%, respectively) [100], whereas DHA is prominently accumulated in most *Schizochytriumm* sp. (37.10–63.1%) (GRAS notice (GRN) no. 677 and 844)*, Aurantiochytrium* (30–40%) [101], *Crypthecodinium* (40–45%) [102], and *Ulkenia* sp. (45%) [103] with the presence of trace amounts of EPA (Table 1).

In *Aurantiochytrium limacinum* SR2, DHA can accumulate up to 48.51% of the total FAs, with high productivity (32.36 g/L and 337.1 mg/L/h) under fed-batch fermentation [104]. *Schizochytrium limacinum* SR21 produced DHA contents of 45.54 and 67.76% of total lipids in flask and bioreactor fermentation, respectively [105]. When glycerol is used as a carbon source, *Thraustochytrium* sp. ONC T18 can accumulate 36.86 of oil (dry weight; 11.67 g/L of culture) rich in DHA (37.80%) [106].

Microalgae are the key source of VLC-PUFAs for zooplankton, fish, and other multicellular organisms [10]. With a high percentage of total lipids (up to 37–60% of dry weight), microalgae such as *Nannochloropsis* sp. can accumulate up to 37.8% EPA [107]. Moreover, microalgae are rich in essential amino acids, lipids (in antioxidant fucosterol and β-sitosterol), polysaccharides (e.g., alginate and β-glucans in brown algae), vitamins (including vitamins A, E, B1, B2, B6, and B12), and minerals [10].

With a rapid multiplication and short harvesting time, microalgae are more productive than are other possible sources, including bacteria, fungi, fish, and transgenic plants [9]. In addition, the high energy content of n−3 PUFAs and an ability to maintain membrane fluidity lead to the high accumulation of n−3 PUFAs during stress conditions, such as salinity, temperature, UV- radiation, nutrient depletion, and *p*H [108]. Notably, low-temperature stress is the most important factor for PUFA production, because PUFA (mainly EPA and DHA) helps survival during low-temperature conditions by maintaining the fluidity of the membrane. In *Nannochloropsis* sp., low temperature (10 °C) and low light augmented EPA formation 3.4-fold by shifting the late log phase growth culture [109]. Similarly, in *Phaeodactylum tricornutum*, phosphate depletion, high urea concentration (0.01 M), high CO_2_ levels (0.15%), and decreased in temperature (e.g., from 25 °C to 15 °C) can increase EPA accumulation by 45.0, 38.6, 73, and 18%, respectively [110]. It has been estimated that with a biorefinery setting, *Phaeodactylum tricornutum* microalgae have potential for value generation of n−3-rich oil and high-value protein, with cost estimations in Australian dollars of AUD 20.47 for n−3-rich oil and AUD 6.14 per kg for dry biomass. Moreover, in a biorefinery, n−3 PUFAs can be purified from their lipids, and the remaining fractions can be used for biodiesel production, and the high-value algal biomass can be used as a protein-rich animal feed [9].

Seaweed, or macroalgae, belongs to several species of macroscopic, multicellular, marine algae of Rhodophyta (red), Phaeophyta (brown), and Chlorophyta (green) taxon. VLC-n−3 PUFAs, especially EPA, form the major constituent (32–34% in rhodophytes) of macroalgae lipids [111]. However, most macroalgae contain little lipids (0.85–3.74% DW) [111] and so cannot be a significant source of n−3 PUFAs. However, the consumption of seaweed can provide proteins packed with essential amino acids, health-beneficial carotenoids, and dietary fiber. In macroalgae belonging to chlorophytes, rhodophytes, and phaeophytes, Pereira et al. [112] recorded 9.5–18.0, 2.90–27.26, and 6.57–15.37% of n−3 PUFAs, respectively. In this study, rhodophytes showed the best n−6/n−3 PUFAs ratio of 0.60–1.92, and chlorophytes and phaeophytes showed n−6/n−3 ratios of 0.31–31.25 and 2.28–3.89, respectively. Among the 17 macroalgae belonging to different phyla investigated, *Pterocladiella capillacea* (Rhodophyta) showed the best n−6/n−3 PUFAs ratio of 0.91, and the lowest n−6/n−3 PUFAs ratio of 0.29 was recorded in *Bornetia secundiflora* (Rhodophyta), because the proportions were high for n−3 PUFAs (27.26%) and low for n−6 PUFAs (7.94%). 

Among the brown, green, and red macroalgae species from North Queensland, Australia, the highest amount of EPA (3.30 mg/g DW) was recorded from red seaweeds (*Champia parvula*) [113]. In another study, among the Norwegian seaweed species, the highest content of EPA was recorded from red seaweed, accounting for 32.1% and 34.3% of the total FAs in *Vertebrata lanosa* and *Palmaria palmata*, respectively [111].

**Table 1:** n−3 and n−6 fatty-acid content in the selected plant and animal-based foods.

	Source	Oil Content (%)	ALA	SDA	EPA	DHA	n−6/n−3	Reference
Seeds	*Brassica napus* sp. *oleifera* L. (rapeseed/canola)	36.9–40.5	8.7–9.5				1.9–2.5	[56]
	*Buglossoides arvensis* (L.) I.M. Johnst. (Corn gromwell; Ahiflower^®^) *	20.0	49.6	21			0.18	[92]
	*Camelina sativa* (L.) Crtz. (Camelina) *	29.6–49.0	19.1–43.1					[71]
	*Echium canatabricum*		33.6	14.7			0.55	[91]
	*Echium plantagineum* (Purple viper’s bugloss)	24.1	34.5	11.0			0.6	[90]
	*Eucommia ulmoides* Oliver	34.63	61.36					[82]
	*Lappula patula*		40.0	13.6			0.40	[91]
	*Lepidium sativum* (Garden cress)	21–24	30.34				0.42	[114]
	*Linum usitatissimum* (Flax)	38.76	53.4				0.290	[60]
	*Mertensia alpine* (Torr.) G.Don.	-	12.9	9.3			1.6	[115]
	*Mertensia ciliata* (James ex Torr.) G. Don.	-	11.8	6.4			1.6	[115]
	*Ocimum basilicum* (Basil)	22.0	63.8				0.320	[60]
	*Paeonia* section *Moutan* DC. (Tree peony)		26.1–54.7				0.4–1.0	[75]
	*Perilla frutescens* (Perilla)	42.8	65.6				0.190	[60]
	*Plukenetia volubilis* L. (Sacha inchi)	33.4–37.6	37.3–44.2				0.83–1.09	[77]
	*Ribes nidrigolaria* Bauer (Jostaberry)		28.01	5.45	-	-	1.17	[94]
	*Ribes nigrum* L. (Blackcurrant)		14.89	2.86	-	-	3.17	[94]
	*Ribes rubrum* L. (Redcurrant)		24.40	3.35	-	-	1.48	[94]
	*Ribes uva-crispa* L. (Gooseberry)		20.54	4.32	-	-	1.82	[94]
	*Salvia hispanica* L. (Chia) *	30.17–32.16	54.5–64.7					[68]
Herb	*Mertensia maritima* (L.) Gray	10.9	30.4	6			0.85	[95]
	*Portulaca oleracea* L. (Purslane)		45.30–51.2				1:1–1:3	[85]
Microalgae	*Isochrysis galbana*		3.1			11.8		[116]
	*Nannochloropsis salina* *				25–30			http://www.lyxia.com/product/ [Accessed 14 Oct 2021]
	*Nannochloropsis* sp.	37–60	0.1–17.5		4.7–33.7			[107]
	*Nannochloropsis* sp. CCNM 1081	39.8			27.6			[109]
	*Nannochloropsis* sp. BR2		0.4		18.8			[116]
	*Pavlova lutheri*		0.1		21.8			[116]
	*Phaeodactylum tricornutum* Bohlin		0.38–0.40	0.87–1.14	22.8–30.7	0.98–1.70		[117]
Thraustochytrid	*Aurantiochytrium limacinum* SR21					30–40		[101]
	*Crypthecodinium cohnii* *					40–45		[102]
	*Schizochytrium* sp.			0.07	16.2	33.7		[100]
	*Schizochytrium limacinum SR21*	52.3				66.7		[118]
	*Thraustochytrium* sp. ONC T18					37.8		[106]
	*Schizochytrium* sp. FCC-3204		0.1	0.3–0.4	0.5-0.9	59.8–63.1		GRAS Notice (GRN) No. 844
	*Schizochytrium* sp. ONC-Tl8 *			0.20–0.32		37.10–42.47		GRAS Notice (GRN) No. 677
	*Ulkenia* sp. SAM 2179 *					45		[103]
Seaweeds	*Codium fragile* (Suhr) Hariot (Chlorophyta)	2.7	14.2–19.9		3.0–4.4		0.3	[119]
	*Laminaria digitata* (Hudson) J.V. Lamouroux (Phaeophyceae)	~1.5	5.0–5.5		12.5–13.1		0.5–0.7	[119]
	*Palmaria palmata*	1.39	0.8		32.1		0.4	[111]
	*Palmaria palmata* (L.) O. Kuntze (Rhodophyta)	~1.5	1.5		36.8–41.2		0.1	[119]
	*Vertebrata lanosa*	1.8			34.3		0.03	[111]

* Commercially exploited sources for n−3 PUFAs.

### 5.5. Genetically Modified (GM) Plants

In the past decade, significant progress has been made in the successful reconstitution of the LC-PUFA biosynthetic pathway in oilseed crops, triggering their substantial accumulation in the seeds [120,121]. To date, several GM oilseed crops producing GLA and SDA have been authorized for use in food products. For instance, GM soybean line MON87769 expressing Δ15-desaturase (from *Neurospora crassa*), which converts LA to ALA, and Δ6-desaturase (*Primula juliae*), responsible for the conversion ALA to SDA, has been authorized for food and feed use by Australia, the European Union, Japan, Canada, Korea, and other countries (OECD Unique Identifier MON-87769-7). GM canola expressing Δ12-desaturase from *Lachancea kluyveri*, Δ15-desaturase from *Pichia pastoris*, Δ6-desaturase from *Micromonas pusilla*, Δ6-elongase from *Pyramimonas cordata*, Δ5-desaturase from *Pavlova salina*, Δ5-elongase from *Pyramimonas cordata*, and Δ4-desaturase from *Pavlova salina* has been developed to produce DHA, and has been authorized for food and feed use by Australia (OECD Unique Identifier NS-B5ØØ27-4). Growing demand for omega-3-PUFAs has led to the development of transgenic plants to produce de novo terrestrial sources of EPA and DHA. Different research groups have successfully demonstrated the production of EPA and DHA in traditional oilseeds crops such as camelina by metabolic engineering [122,123]. Recently, large-scale field evaluation of transgenic camelina plants expressing different combinations of desaturases and elongases from plants and microalgae has shown they accumulate 15–20% of EPA + DHA [124]. However, the oxidative stability of these PUFA-rich seed oils is a bottleneck for their commercial exploitation. 

Metabolically engineered food-grade yeast *Yarrowia lipolytica* was also reported to produce more than 25% EPA of its dry cell weight (DCW) under commercial-scale fermentation [125]. *Y. lipolytica* can be considered to be a viable source of EPA production; its cell density of 100 g DCW/L of the fermentation medium can yield 25 g EPA, with high lipid productivity of 1 g/L/h [125]. Wild type *Y. lipolytica* accumulates mainly oleic acid and linoleic acid (primarily TAG forms), which can be diverted to EPA production (more than 50% of total FAs) via Δ9/Δ8 pathway engineering. 

## 6. Low Oxidative Stability of PUFAs in Foods Is a Challenge

Lipids are susceptible to oxidation that generates unsaturated carbonyls, harmful reaction products, and undesirable flavor during extraction, storage, and food preparations [126]. PUFAs are more susceptible to chemical modifications, such as metal-catalyzed autoxidation and hydrogenation, than are SFAs, because they possess multiple C=C bonds that are vulnerable to electrophilic attack. Oxidative loss of PUFAs in foods and supplements is generally controlled by adding natural antioxidants (e.g., plant extract), synthetic antioxidants (e.g., DL-α-tocopherol, butylated hydroxytoluene (BHT), butylated hydroxyanisole (BHA), and ascorbyl palmitate (AP)), or metal chelating agents (e.g., phytic acid) alone or in combination [127,128,129,130].

Natural (D-α-) and synthetic (DL-α-) forms of tocopherol (vitamin E) are widely used as additives in food and food supplements [131]. A commercial mix of ALA, EPA, and DHA (Dry n−3^®^ 5:25 C) by BASF SE (Ludwigshafen, Germany) is sold as a gelatin capsule with added DL-α-tocopherol as an antioxidant. Antioxidants inhibit oxidative degradation by reducing the rate of oxidation or delaying the induction of autoxidation by scavenging the lipid peroxides and free radicals or controlling transition metals.

In recent years, with increasing consumer preference for natural over synthetic products, several investigations have attempted to incorporate natural antioxidants (e.g., polyphenols, carotenoids, and tocopherols) from the edible plant materials [127,128,129,130]. Polyphenol-rich rosemary extracts have been shown as more efficient than α-tocopherol BHT in stabilizing the n−3 PUFA in flaxseed oil [127]. Similarly, catechin-rich green tea extract is more effective than α-tocopherol in stabilizing DHA-rich oil [129]. The carotenoid-, tocopherol-, and polyphenol-rich extracts from edible flowers have also shown oxidative protection of cold-pressed flax and chia seed oils [130]. 

Micro- and nano-encapsulation is a promising approach to protecting core material (PUFAs) from environmental factors, such as oxygen, light, and transition metals, thus improving their oxidative stability and bioactivities [132]. The wall material used for microencapsulation and methods used for microencapsulation of n−3 fatty acids are widely reviewed [133,134,135,136,137,138]. Currently, several vegan n−3 PUFA-based microencapsulated products are available commercially (Table 2). Friesland Campina N.V. (Amersfoort, Netherlands) offers microencapsulated LC-PUFA, including microalgal-derived DHA (Vana^®^-Sana algae DHA 11 IF), which provides PUFAs with good nutritional and sensorial quality. The vegan-grade microencapsulated algal oil powder betamega³ (120 mg DHA/g) and Gamma³ algal Omega-3 DHA emulsions (400 mg DHA/g emulsion) are marketed by Algarithm Ingredients, Inc. (Saskatoon, Saskatchewan). Cubiq Foods (Granollers. Barcelona) is currently marketing the Go!Mega3^®^ a micro-encapsulated (30–50 µ in size) n−3-enriched algae oil with 2% DHA+EPA (*w*/*w*). Seanova (Finistère, Brittany) offers Algal DHA powder H100 (100 mg/g DHA from *Schizochytrium* sp.), chia powder-125 (60 mg/g ALA), and chia powder-435 (55 mg/g ALA).

## 7. Emulsion-Based PUFAs Can Be Used in Beverages

Adding lipophilic ingredients such as PUFAs to liquid products is challenging, because of their insolubility in water. Colloidal-based delivery systems, including filled hydrogel particles, emulsions, and multilayer emulsions, can effectively incorporate PUFAs into aqueous environments, without interfering with the turbidity or opacity. Moreover, these systems can significantly improve the oxidative stability and bioaccessibility of PUFAs [139,140]. Emulsion-based VLC n−3 PUFA (e.g., emulsified algal DHA oil) has received GRAS status (GRAS Notice No. GRN 000621). In recent years, the development of PUFA oil emulsions has increased considerably; as a result, several fish-oil PUFAs and some vegan PUFA emulsion-based ingredients are commercially available to use in food fortification. OceansOmega (New York, USA; Mycell Technologies company) provides GRAS and Kosher-certified water-soluble and stabilized DHA (OTEC™ D-3500-A; 3.6% emulsion) from a microalgal source for use in fortified water, juices, carbonated drinks, and many more food products. DSM Nutritional Products, Inc. (Heerlen, Netherlands) offers several marine n−3 PUFA-based ingredients, including MEG-3^®^ ‘15’ n−3 Emulsion LV (min. 6.25% DHA and 4.5% EPA) to use in dietary supplements.

## 8. Bioavailability of Vegan n−3 PUFAs: Algal-Oil Supplements Are a Viable Alternative to Fish Oil

FAs may be present in the body as free FAs, bound to glycerol, to form monoacylglycerol (MAG), diacylglycerol (DAG), or triacylglycerol (TAG), or bound to membrane phospholipids (PL). The bioavailability of FAs depends on the lipid form and can be ranked as PL > TG > FFA [141]. Moreover, the food matrices and structures of FAs bound to PLs and TAGs (e.g., *sn*-1, *sn*-2, or *sn−3* positions) can also influence the bioavailability and their distribution in the body [2,142]. The FAs occurring in the *sn*-2 position escape from the pancreatic and lipoprotein lipases-mediated hydrolysis and are more readily absorbed in the body [143]. Thus, PUFAs with *sn*-2 position are considered a more effective dietary source [143,144]. In plants, microalgae, thraustochytrids, fish, and krill PUFAs are generally found in TAG form bound to the *sn*-2 position [142,144,145]. In contrast, seal (marine mammal) oil PUFAs are primarily bound to the *sn*-1 position of TAG [2,144].

The RBC EPA + DHA (n−3 index) is usually considered to be a good indicator of long-term bioavailability of VLC-n−3 PUFAs, and the levels in RBCs reflect those of other tissues, including hepatic, myocardial, and nephritic tissues [4,141,142]. Most importantly, RBC EPA + DHA levels provide unbiased predictive information for a variety of diseases and deliver valuable information in the VLC-n−3 PUFAs status screening [4].

Much research has examined subjects’ n−3PUFA status after eating plant or algal-derived n−3 PUFAs (ALA, SDA, EPA, and DHA). Most studies have suggested that plant (e.g., echium and linseed oil, garden cress oil) or algal-derived n−3 PUFAs can successfully be used to increase blood levels of EPA + DHA [146,147,148,149,150], whereas some studies found no benefits after plant/algal derived n−3 PUFAs [151].

In the bioavailability studies, plant SDA-based oil (e.g., echium oil) have shown more beneficial effects in increasing the blood EPA + DHA than has plant-based ALA-rich oil (e.g, linseed oil) [147], most probably because of the rate-limiting step of bioconversion of ALA to SDA. In a double-blind, randomized intervention study, echium oil supplementation (2g SDA/d) for 10 weeks was substantially more beneficial in increasing the plasma and erythrocyte EPA than was linseed oil (7 g ALA/d), and microalgae oil (DHA 2 g/d) was beneficial for increasing DHA levels [147]. In another double-blind, parallel-arm, randomized controlled study, participants (*n* = 80, age groups of 20–35 and 49–69 years) who were administered echium oil (5 g of ALA, 2 g of SDA; *n* = 59) for 8 weeks had increased plasma and peripheral blood mononuclear cell (PBMCs), ALA, SDA, EPA (168% and 79%, respectively), and DPA (68% and 39%, respectively) but decreased DHA (−5% and −23%, respectively) [146]. In contrast, fish-oil supplementation (1.9 g EPA/d; *n* = 19) demonstrated a 533% and 497% increase in EPA in plasma and PBMC, respectively, whereas DHA remained unchanged [146].

Because algal oils can directly supply EPA and DHA (like fish oil), they are most valuable in improving the body status of EPA + DHA [148,149,150]. A double-blind, randomized, placebo-controlled trial of pregnant women provided with 400 mg/d algal DHA for 20 weeks of singleton gestation through 6 months postpartum showed significantly increased RBC phospholipids DHA (1.94 mol % of fatty acid) compared to the placebo group at delivery (0.84 mol % of fatty acid) [150]. A double-blind, parallel trial comprising 93 healthy adults with hypertriglyceridemia demonstrated that algal oil (2.4 g/d DHA and EPA in a 2.7:1 ratio) and fish oil (2.0 g/day DHA and EPA in a 0.7:1 ratio) are equally beneficial in decreasing TAG levels (−18.9, −22.9%, respectively). The algal oil was more beneficial in increasing the plasma levels of DHA, and fish oil more advantageous in increasing the EPA levels after 14 weeks of supplementation, probably because of the high contents of DHA in algal oil and EPA in fish oil. Supplementation with 600 mg/d of DHA from either fish-oil capsules or algal-oil capsules for 2 weeks demonstrated a significant increase in plasma DHA levels of 71.60–84.22 µg/mL in the vegetarian/vegan group in spite of lower levels of plasma DHA at baseline (34.10 µg/mL) compared to the omnivorous/fish eaters group, and finally all groups ended with similar levels of 71.14–93.23 µg/mL DHA [149]. These findings suggest that in terms of DHA, algal-oil supplements are a viable alternative to fish-oil supplements for omnivores, vegans, and vegetarians.

## 9. Commercial Availability of Plant and Microbial-Based n−3 PUFAs

Most plant- and microbial-based-sources of n−3 PUFA discussed in this review are currently available as commercial formulations. The vegan n−3 PUFA-based microencapsulated products are listed in Table 2. The other major vegan n−3 PUFA-based commercially available dietary products are listed in Table 3.

## 10. Conclusions and Future Perspectives

Omega-3 fatty acids are essential for normal growth and development but also have positive effects on the heart, brain, eyes, joints, skin, mood, and behavior. EPA, associated with the hydrocarbon core of the lipid membrane, efficiently inhibits the propagation of free radicals, thus preventing lipid (e.g., LDL) oxidation. In contrast, DHA-derived specialized SPMs (e.g., protectins, resolvins, and maresins) are critically important for neuroprotection. With these and other vital functions of EPA and DHA in cellular protection, the higher availability of these key VLC-n−3 PUFAs is potentially beneficial in cardiovascular, neurodegenerative (e.g., bipolar disorder and cognitive impairment), and several other chronic diseases.

The bioavailability studies suggest that, to maintain availability of EPA and DHA, a diet rich in EPA and DHA is most beneficial, followed by SDA, whereas ALA is least beneficial, probably because of the very low bioconversion rate. However, for vegans and vegetarians who do not consume SDA, EPA, and DHA-rich supplements, ALA is the only source for EPA and DHA in the body. Among such populations, oils used for cooking (especially canola oil) are the most common source of ALA. Moreover, chia, flax, camelina, garden cress seeds, or seed oil in low-heat cooking are the most viable sources for enriching the diet with ALA. Most of the plant species that produce nutritionally important fatty acids (ALA and SDA), such as *Buglossoides arvensis*, *Echium* sp., *Perilla frutescens*, and sacha inchi, are wild and not agronomically adapted. Targeted research focusing on the development of agronomic practices of the above crops in different geographical conditions may help in meeting the ever-increasing demand for vegan n−3 sources. In recent years, GM oilseeds crops, soybean, and canola that produce SDA have been developed and authorized for use in food products. In the future, cooking oil from these crops may contribute significantly to supplying the SDA for the daily diet. Alternatively, future research should focus on the development of vegan food products enriched with PUFAs without compromising oxidative stability. Incorporation of vegan PUFA-rich seed oils in food products, such as margarine, salads, mayonnaise, smoothies, pastries, ice creams, and breakfast bars, may improve their bioavailability and oxidative stability.

In the most viable approach, oil from microalgae, such as *Nannochloropsis* sp., and thraustochytrids, such as *Aurantiochytrium limacinum*, *Crypthecodinium cohnii*, *Schizochytrium* sp., and *Ulkenia* sp., can directly supply a significant amount of EPA and DHA. Thus, these microbial sources are currently used for the commercial production of vegan EPA and DHA.

Low oxidative stability of PUFAs in foods is a challenge, and it is generally controlled by adding synthetic antioxidants (e.g., DL-α-tocopherol). However, with increasing consumer preference for natural over synthetic products, investigations attempting to incorporate natural antioxidants (e.g., polyphenols, carotenoids, and tocopherols) from edible plant materials have shown promising results. 

Microencapsulation is a promising technique to protect PUFAs from auto-oxidation caused by environmental effects, thus improving their oxidative stability. However, the microencapsulation of PUFA-rich oil changes its physical state from liquid to powder. Thus, more promising and innovative approaches are needed to protect PUFAs in the extracted oil. On the other hand, biofortification of PUFA-rich seeds with natural antioxidants, such as tocols (tocopherols and tocochromanols), may increase the oxidative stability of the oils. Hence, future research should explore the possibility of metabolic engineering strategies to increase natural antioxidant levels in PUFA-rich oil-seed crops.

Most vegan-based sources of n−3 PUFA discussed in this review are currently available as commercial formulations. For instance, canola, chia, flax, camelina, perilla, garden cress seeds, and seed oil are commercially available foods rich in ALA. Similarly, *B. arvensis* and *E. plantagineum* seed oils rich in SDA are commercially available for culinary uses. Moreover, several microalgae-based dietary supplements such as DHASCO-B^®^ nutritional oil and powder and life’sDHA^®^ vegetarian capsules are a popular commercially available source of EPA and DHA. The adequate consumption of these n−3 PUFA-rich foods and dietary supplements may improve health. 

## Figures and Tables

**Table 2:** Microencapsulated vegan n−3 fatty acids-based commercial products.

Company	Ingredient Brand	Major n−3 Fatty Acids
FrieslandCampina N.V. (Amersfoort, Netherlands)	Vana^®^-Sana algae DHA 11 IF	Microalgal derived DHA
Algarithm Ingredients, Inc. (Saskatoon, Saskatchewan)	Betamega³	Microalgal oil powder (120 mg DHA/g)
	Gamma³	Microalgal DHA emulsions (400 mg DHA/g emulsion)
Cubiq Foods (Granollers. Barcelona)	Go!Mega3^®^	Microalgal DHA+EPA (2% *w*/*w*)
Seanova (Finistère, Brittany)	Algal DHA powder H100	100 mg/g DHA from *Schizochytrium* sp.
	Chia powder-125	60 mg/g ALA from chia seeds
	Chia powder-435	55 mg/g ALA from chia seeds

Source: Company websites.

**Table 3:** The major commercially available vegan n−3 PUFA-based dietary products.

n−3 PUFA	Commercially Available Product	Dietary Uses	Reference
ALA	Canola seed oil	Vegetable oil	[152]
	Flax seeds and seed oil	Baked goods, juices, dairy products, and dry pasta products	[59]
	Chia seeds and seed oil	Baked and dairy products as emulsifier and stabilizer	[68,69]
	Camelina seed oil	Edible oil, food supplement	[153]
	Garden cress seed oil	Salad dressings	[154]
	Perilla seed oil	Edible oil	[60]
	Purslane plant (stems and leaves)	Green leafy vegetable	[85]
SDA	*Echium plantagineum* oil	Salad dressings/smoothies	[90]
	*Buglossoides arvensis* oil *(*Ahiflower^®^ oil)	Oil for salad dressing, soft-gel capsules for dietary supplement	[45]
	Foliage of *Mertensia maritima*	Culinary recipes	[155]
EPA	Microaglage *Nannochloropsis* sp.	Dietary supplement	http://jemmaxnutraceuticals.com/ [Accessed 14 Oct 2021]
DHA	DHASCO-B^®^ nutritional oil and powder, and life’sDHA^®^ vegetarian capsules from microalgae	Dietary supplement	https://www.dsm.com/ [Accessed 14 Oct 2021]
	Vegan Omega 3 DHA capsules from Thraustochytrid *Schizochytrium* sp.	Dietary supplement	https://omvits.com/ [Accessed 14 Oct 2021]; https://www.naturopathica.com.au/ [Accessed 14 Oct 2021]

## Data Availability

Data is contained within the article.

## Abbreviations

ALA	α-Linolenic acid
AP	Ascorbyl palmitate
ARA	Arachidonic acid
BHA	Butylated hydroxyanisole
BHT	Butylated hydroxytoluene
CHD	Coronary heart disease
CVD	Cardiovascular diseases
DAG	Diacylglycerol
DHA	Docosahexaenoic acid
DPA	Docosapentaenoic acid
EPA	Eicosapentaenoic acid
GCO	Garden cress oil
GLA	γ-Linolenic acid
GM	Genetically modified
GRAS	Generally recognized as safe
LA	Linoleic acid
LDL	Low-density lipoprotein
MAG	Monoacylglycerol
MUFAs	Monounsaturated fatty acids
PBMCs	Peripheral blood mononuclear cell
PLs	Phospholipids
RBC	Red blood cells
SDA	Stearidonic acid
SPM	Pro-resolving mediators
TAG	Triacylglycerol
VLC-PUFAs	Very long chain-poly unsaturated fatty acids

## References

[1] Bazinet R.P., Layé S. (2014). Polyunsaturated fatty acids and their metabolites in brain function and disease. Nat. Rev. Neurosci..

[2] Saini R.K., Keum Y.S. (2018). Omega-3 and omega-6 polyunsaturated fatty acids: Dietary sources, metabolism, and significance—A review. Life Sci..

[3] Johnson G.H., Fritsche K. (2012). Effect of dietary linoleic acid on markers of inflammation in healthy persons: A systematic review of randomized controlled trials. J. Acad. Nutr. Diet.

[4] Harris W.S. (2018). The Omega-6:Omega-3 ratio: A critical appraisal and possible successor. Prostaglandins Leukot. Essent. Fat. Acids.

[5] Appleby P.N., Key T.J. (2016). The long-term health of vegetarians and vegans. Proc. Nutr. Soc..

[6] Craig W.J., Mangels A.R. (2009). Position of the American Dietetic Association: Vegetarian diets. J. Am. Diet. Assoc..

[7] Sarter B., Kelsey K.S., Schwartz T.A., Harris W.S. (2015). Blood docosahexaenoic acid and eicosapentaenoic acid in vegans: Associations with age and gender and effects of an algal-derived omega-3 fatty acid supplement. Clin. Nutr..

[8] Burns-Whitmore B., Froyen E., Heskey C., Parker T., San Pablo G. (2019). Alpha-Linolenic and Linoleic Fatty Acids in the Vegan Diet: Do They Require Dietary Reference Intake/Adequate Intake Special Consideration?. Nutrients.

[9] Adarme-Vega T., Lim D.K.Y., Timmins M., Vernen F., Li Y., Schenk P.M. (2012). Microalgal biofactories: A promising approach towards sustainable omega-3 fatty acid production. Microb. Cell Factories.

[10] Wells M.L., Potin P., Craigie J.S., Raven J.A., Merchant S.S., Helliwell K.E., Smith A.G., Camire M.E., Brawley S.H. (2017). Algae as nutritional and functional food sources: Revisiting our understanding. J. Appl. Phycol..

[11] Orozco Colonia B.S., Vinícius de Melo Pereira G., Soccol C.R. (2020). Omega-3 microbial oils from marine thraustochytrids as a sustainable and technological solution: A review and patent landscape. Trends Food Sci. Technol..

[12] Burdge G.C. (2006). Metabolism of α-linolenic acid in humans. Prostaglandins Leukot. Essent. Fat. Acids.

[13] Botelho P.B., Mariano K.D.R., Rogero M.M., De Castro I.A. (2013). Effect of Echium oil compared with marine oils on lipid profile and inhibition of hepatic steatosis in LDLr knockout mice. Lipids Health Dis..

[14] Schwab U.S., Callaway J.C., Erkkilä A.T., Gynther J., Uusitupa M.I.J., Järvinen T. (2006). Effects of hempseed and flaxseed oils on the profile of serum lipids, serum total and lipoprotein lipid concentrations and haemostatic factors. Eur. J. Nutr..

[15] Yue H., Qiu B., Jia M., Liu W., Guo X.-F., Li N., Xu Z.-X., Du F.-L., Xu T., Li D. (2020). Effects of α-linolenic acid intake on blood lipid profiles: A systematic review and meta-analysis of randomized controlled trials. Crit. Rev. Food Sci. Nutr..

[16] Sherratt S.C.R., Mason R.P. (2018). Eicosapentaenoic acid and docosahexaenoic acid have distinct membrane locations and lipid interactions as determined by X-ray diffraction. Chem. Phys. Lipids.

[17] Saenz de Viteri M., Hernandez M., Bilbao-Malavé V., Fernandez-Robredo P., González-Zamora J., Garcia-Garcia L., Ispizua N., Recalde S., Garcia-Layana A. (2020). A Higher Proportion of Eicosapentaenoic Acid (EPA) When Combined with Docosahexaenoic Acid (DHA) in Omega-3 Dietary Supplements Provides Higher Antioxidant Effects in Human Retinal Cells. Antioxidants.

[18] Belayev L., Hong S.H., Menghani H., Marcell S.J., Obenaus A., Freitas R.S., Khoutorova L., Balaszczuk V., Jun B., Oriá R.B. (2018). Docosanoids Promote Neurogenesis and Angiogenesis, Blood-Brain Barrier Integrity, Penumbra Protection, and Neurobehavioral Recovery After Experimental Ischemic Stroke. Mol. Neurobiol..

[19] Mason R.P., Sherratt S.C.R., Jacob R.F. (2016). Eicosapentaenoic Acid Inhibits Oxidation of ApoB-containing Lipoprotein Particles of Different Size In Vitro When Administered Alone or in Combination With Atorvastatin Active Metabolite Compared With Other Triglyceride-lowering Agents. J. Cardiovasc. Pharmacol..

[20] Mayurasakorn K., Niatsetskaya Z.V., Sosunov S.A., Williams J.J., Zirpoli H., Vlasakov I., Deckelbaum R.J., Ten V.S. (2016). DHA but Not EPA Emulsions Preserve Neurological and Mitochondrial Function after Brain Hypoxia-Ischemia in Neonatal Mice. PLoS ONE.

[21] Ohnishi H., Saito Y. (2013). Eicosapentaenoic Acid (EPA) Reduces Cardiovascular Events: Relationship with the EPA/Arachidonic Acid Ratio. J. Atheroscler. Thromb..

[22] Mason R.P., Libby P., Bhatt D.L. (2020). Emerging Mechanisms of Cardiovascular Protection for the Omega-3 Fatty Acid Eicosapentaenoic Acid. Arterioscler. Thromb. Vasc. Biol..

[23] Ghasemi Fard S., Wang F., Sinclair A.J., Elliott G., Turchini G.M. (2019). How does high DHA fish oil affect health? A systematic review of evidence. Crit. Rev. Food Sci. Nutr..

[24] Lafuente M., Rodríguez González-Herrero M.E., Romeo Villadóniga S., Domingo J.C. (2021). Antioxidant Activity and Neuroprotective Role of Docosahexaenoic Acid (DHA) Supplementation in Eye Diseases That Can Lead to Blindness: A Narrative Review. Antioxidants.

[25] Del Gobbo L.C., Imamura F., Aslibekyan S., Marklund M., Virtanen J.K., Wennberg M., Yakoob M.Y., Chiuve S.E., dela Cruz L., Frazier-Wood A.C. (2016). ω-3 Polyunsaturated Fatty Acid Biomarkers and Coronary Heart Disease: Pooling Project of 19 Cohort Studies. JAMA Intern. Med..

[26] Delgado-Lista J., Perez-Martinez P., Lopez-Miranda J., Perez-Jimenez F. (2012). Long chain omega-3 fatty acids and cardiovascular disease: A systematic review. Br. J. Nutr..

[27] Mozaffarian D., Lemaitre R.N., King I.B., Song X., Huang H., Sacks F.M., Rimm E.B., Wang M., Siscovick D.S. (2013). Plasma Phospholipid Long-Chain ω-3 Fatty Acids and Total and Cause-Specific Mortality in Older Adults. Ann. Intern. Med..

[28] Deckelbaum R.J., Calder P.C. (2020). Editorial: Is it time to separate EPA from DHA when using omega-3 fatty acids to protect heart and brain?. Curr. Opin. Clin. Nutr. Metab. Care.

[29] Bhatt D.L., Steg P.G., Miller M., Brinton E.A., Jacobson T.A., Ketchum S.B., Doyle R.T., Juliano R.A., Jiao L., Granowitz C. (2018). Cardiovascular Risk Reduction with Icosapent Ethyl for Hypertriglyceridemia. N. Engl. J. Med..

[30] Simopoulos A. (2016). An Increase in the Omega-6/Omega-3 Fatty Acid Ratio Increases the Risk for Obesity. Nutrients.

[31] Grosso G., Pajak A., Marventano S., Castellano S., Galvano F., Bucolo C., Drago F., Caraci F. (2014). Role of Omega-3 Fatty Acids in the Treatment of Depressive Disorders: A Comprehensive Meta-Analysis of Randomized Clinical Trials. PLoS ONE.

[32] Yates C.M., Calder P.C., Ed Rainger G. (2014). Pharmacology and therapeutics of omega-3 polyunsaturated fatty acids in chronic inflammatory disease. Pharmacol. Ther..

[33] Yan Y., Jiang W., Spinetti T., Tardivel A., Castillo R., Bourquin C., Guarda G., Tian Z., Tschopp J., Zhou R. (2013). Omega-3 Fatty Acids Prevent Inflammation and Metabolic Disorder through Inhibition of NLRP3 Inflammasome Activation. Immunity.

[34] Swanson D., Block R., Mousa S.A. (2012). Omega-3 Fatty Acids EPA and DHA: Health Benefits Throughout Life. Adv. Nutr..

[35] Wu J.H.Y., Micha R., Imamura F., Pan A., Biggs M.L., Ajaz O., Djousse L., Hu F.B., Mozaffarian D. (2012). Omega-3 fatty acids and incident type 2 diabetes: A systematic review and meta-analysis. Br. J. Nutr..

[36] Abdelhamid A.S., Brown T.J., Brainard J.S., Biswas P., Thorpe G.C., Moore H.J., Deane K.H., Summerbell C.D., Worthington H.V., Song F. (2020). Omega-3 fatty acids for the primary and secondary prevention of cardiovascular disease. Cochrane Database Syst. Rev..

[37] Lange K.W., Nakamura Y., Gosslau A.M., Li S. (2019). Are there serious adverse effects of omega-3 polyunsaturated fatty acid supplements?. J. Food Bioact..

[38] Nogueira M.S., Scolaro B., Milne G.L., Castro I.A. (2019). Oxidation products from omega-3 and omega-6 fatty acids during a simulated shelf life of edible oils. LWT.

[39] Zaloga G.P. (2021). Narrative Review of n-3 Polyunsaturated Fatty Acid Supplementation upon Immune Functions, Resolution Molecules and Lipid Peroxidation. Nutrients.

[40] Burdge G.C., Jones A.E., Wootton S.A. (2002). Eicosapentaenoic and docosapentaenoic acids are the principal products of α-linolenic acid metabolism in young men. Br. J. Nutr..

[41] Childs C.E., Kew S., Finnegan Y.E., Minihane A.M., Leigh-Firbank E.C., Williams C.M., Calder P.C. (2014). Increased dietary α-linolenic acid has sex-specific effects upon eicosapentaenoic acid status in humans: Re-examination of data from a randomised, placebo-controlled, parallel study. Nutr. J..

[42] Harnack K., Andersen G., Somoza V. (2009). Quantitation of alpha-linolenic acid elongation to eicosapentaenoic and docosahexaenoic acid as affected by the ratio of n6/n3 fatty acids. Nutr. Metab..

[43] Plourde M., Cunnane S.C. (2007). Extremely limited synthesis of long chain polyunsaturates in adults: Implications for their dietary essentiality and use as supplements. Appl. Physiol. Nutr. Metab..

[44] Brenna J.T., Salem N., Sinclair A.J., Cunnane S.C. (2009). α-Linolenic acid supplementation and conversion to n-3 long-chain polyunsaturated fatty acids in humans. Prostaglandins Leukot. Essent. Fat. Acids.

[45] Cumberford G., Hebard A. (2015). Ahiflower oil: A novel non-GM plant-based omega-3+6 source. Lipid Technol..

[46] Simopoulos A.P. (2006). Evolutionary aspects of diet, the omega-6/omega-3 ratio and genetic variation: Nutritional implications for chronic diseases. Biomed. Pharmacother..

[47] Schulze M.B., Minihane A.M., Saleh R.N.M., Risérus U. (2020). Intake and metabolism of omega-3 and omega-6 polyunsaturated fatty acids: Nutritional implications for cardiometabolic diseases. Lancet Diabetes Endocrinol..

[48] FSA Panel on Dietetic Products, Nutrition, and Allergies (NDA) (2010). Scientific Opinion on Dietary Reference Values for fats, including saturated fatty acids, polyunsaturated fatty acids, monounsaturated fatty acids, trans fatty acids, and cholesterol. EFSA J..

[49] WHO (2003). WHO Technical Report Series 916.

[50] Thompson M., Hein N., Hanson C., Smith L.M., Anderson-Berry A., Richter C.K., Stessy Bisselou K., Kusi Appiah A., Kris-Etherton P., Skulas-Ray A.C. (2019). Omega-3 Fatty Acid Intake by Age, Gender, and Pregnancy Status in the United States: National Health and Nutrition Examination Survey 2003–2014. Nutrients.

[51] Harris W.S., von Schacky C. (2004). The Omega-3 Index: A new risk factor for death from coronary heart disease?. Prev. Med..

[52] Harris W.S. (2009). The omega-3 index: From biomarker to risk marker to risk factor. Curr. Atheroscler. Rep..

[53] Lane K.E., Wilson M., Hellon T.G., Davies I.G. (2021). Bioavailability and conversion of plant based sources of omega-3 fatty acids—A scoping review to update supplementation options for vegetarians and vegans. Crit. Rev. Food Sci. Nutr..

[54] OECD/FAO (2019). OECD-FAO Agricultural Outlook 2019-2028.

[55] Dorni C., Sharma P., Saikia G., Longvah T. (2018). Fatty acid profile of edible oils and fats consumed in India. Food Chem..

[56] Beyzi E., Gunes A., Buyukkilic Beyzi S., Konca Y. (2019). Changes in fatty acid and mineral composition of rapeseed (*Brassica napus* ssp. oleifera L.) oil with seed sizes. Ind. Crop. Prod..

[57] Saini R.K., Rengasamy K.R.R., Ko E.Y., Kim J.T., Keum Y.S. (2020). Korean Maize Hybrids Present Significant Diversity in Fatty Acid Composition: An Investigation to Identify PUFA-Rich Hybrids for a Healthy Diet. Front. Nutr..

[58] Zuk M., Richter D., Matuła J., Szopa J. (2015). Linseed, the multipurpose plant. Ind. Crop. Prod..

[59] Goyal A., Sharma V., Upadhyay N., Gill S., Sihag M. (2014). Flax and flaxseed oil: An ancient medicine & modern functional food. J. Food Sci. Technol..

[60] Zamani Ghaleshahi A., Ezzatpanah H., Rajabzadeh G., Ghavami M. (2020). Comparison and analysis characteristics of flax, perilla and basil seed oils cultivated in Iran. J. Food Sci. Technol..

[61] Marineli R.D.S., Moraes É.A., Lenquiste S.A., Godoy A.T., Eberlin M.N., Maróstica M.R. (2014). Chemical characterization and antioxidant potential of Chilean chia seeds and oil (*Salvia hispanica* L.). LWT—Food Sci. Technol..

[62] Knez Hrnčič M., Ivanovski M., Cör D., Knez Ž. (2019). Chia Seeds (*Salvia Hispanica* L.): An Overview—Phytochemical Profile, Isolation Methods, and Application. Molecules.

[63] Gopalam R., Tumaney A.W. (2021). Functional characterization of acyltransferases from Salvia hispanica that can selectively catalyze the formation of trilinolenin. Phytochemistry.

[64] RV S., Kumari P., Rupwate S.D., Rajasekharan R., Srinivasan M. (2015). Exploring Triacylglycerol Biosynthetic Pathway in Developing Seeds of Chia (*Salvia hispanica* L.): A Transcriptomic Approach. PLoS ONE.

[65] Muñoz L.A., Cobos A., Diaz O., Aguilera J.M. (2013). Chia Seed (*Salvia hispanica*): An Ancient Grain and a New Functional Food. Food Rev. Int..

[66] Ullah R., Nadeem M., Khalique A., Imran M., Mehmood S., Javid A., Hussain J. (2016). Nutritional and therapeutic perspectives of Chia (*Salvia hispanica* L.): A review. J. Food Sci. Technol..

[67] Zúñiga-López M.C., Maturana G., Campmajó G., Saurina J., Núñez O. (2021). Determination of Bioactive Compounds in Sequential Extracts of Chia Leaf (*Salvia hispanica* L.) Using UHPLC-HRMS (Q-Orbitrap) and a Global Evaluation of Antioxidant In Vitro Capacity. Antioxidants.

[68] Melo D., Machado T.B., Oliveira M.B.P.P. (2019). Chia seeds: An ancient grain trending in modern human diets. Food Funct..

[69] Zettel V., Hitzmann B. (2018). Applications of chia (*Salvia hispanica* L.) in food products. Trends Food Sci. Technol..

[70] Campbell M. (2018). Camelina–An alternative oil crop. Biokerosene.

[71] Vollmann J., Eynck C. (2015). Camelina as a sustainable oilseed crop: Contributions of plant breeding and genetic engineering. Biotechnol. J..

[72] Diwakar B.T., Dutta P.K., Lokesh B.R., Naidu K.A. (2010). Physicochemical Properties of Garden Cress (*Lepidium sativum* L.) Seed Oil. J. Am. Oil Chem. Soc..

[73] Umesha S.S., Naidu K.A. (2012). Vegetable oil blends with α-linolenic acid rich Garden cress oil modulate lipid metabolism in experimental rats. Food Chem..

[74] Umesha S.S., Manohar R.S., Indiramma A.R., Akshitha S., Naidu K.A. (2015). Enrichment of biscuits with microencapsulated omega-3 fatty acid (Alpha-linolenic acid) rich Garden cress (*Lepidium sativum*) seed oil: Physical, sensory and storage quality characteristics of biscuits. LWT—Food Sci. Technol..

[75] Li S.-S., Yuan R.-Y., Chen L.-G., Wang L.-S., Hao X.-H., Wang L.-J., Zheng X.-C., Du H. (2015). Systematic qualitative and quantitative assessment of fatty acids in the seeds of 60 tree peony (Paeonia section Moutan DC.) cultivars by GC–MS. Food Chem..

[76] Wang S., Zhu F., Kakuda Y. (2018). Sacha inchi (Plukenetia volubilis L.): Nutritional composition, biological activity, and uses. Food Chem..

[77] Chirinos R., Zuloeta G., Pedreschi R., Mignolet E., Larondelle Y., Campos D. (2013). Sacha inchi (Plukenetia volubilis): A seed source of polyunsaturated fatty acids, tocopherols, phytosterols, phenolic compounds and antioxidant capacity. Food Chem..

[78] Saini R.K., Keum Y.-S., Rengasamy K.R. (2020). Profiling of nutritionally important metabolites in green/red and green perilla (Perilla frutescens Britt.) cultivars: A comparative study. Ind. Crop. Prod..

[79] Prabu S.L., Umamaheswari A., Puratchikody A. (2019). Phytopharmacological potential of the natural gift Moringa oleifera Lam and its therapeutic application: An overview. Asian Pac. J. Trop. Med..

[80] Nazir S., Wani I.A. (2021). Physicochemical characterization of basil (Ocimum basilicum L.) seeds. J. Appl. Res. Med. Aromat. Plants.

[81] Dhama K., Sharun K., Gugjoo M.B., Tiwari R., Alagawany M., Iqbal Yatoo M., Thakur P., Iqbal H.M.N., Chaicumpa W., Michalak I. (2021). A Comprehensive Review on Chemical Profile and Pharmacological Activities of Ocimum basilicum. Food Rev. Int..

[82] Zhang Z.-S., Liu Y.-L., Che L.-M. (2018). Characterization of a New α-Linolenic Acid-Rich Oil: Eucommia ulmoides Seed Oil. J. Food Sci..

[83] Saini R.K., Shetty N.P., Giridhar P. (2014). GC-FID/MS Analysis of Fatty Acids in Indian Cultivars of Moringa oleifera: Potential Sources of PUFA. J. Am. Oil Chem. Soc..

[84] Kim D.E., Shang X., Assefa A.D., Keum Y.S., Saini R.K. (2018). Metabolite profiling of green, green/red, and red lettuce cultivars: Variation in health beneficial compounds and antioxidant potential. Food Res. Int..

[85] Nemzer B., Al-Taher F., Abshiru N. (2020). Phytochemical composition and nutritional value of different plant parts in two cultivated and wild purslane (*Portulaca oleracea* L.) genotypes. Food Chem..

[86] Uddin M.K., Juraimi A.S., Hossain M.S., Nahar M.A., Ali M.E., Rahman M.M. (2014). Purslane weed (Portulaca oleracea): A prospective plant source of nutrition, omega-3 fatty acid, and antioxidant attributes. Sci. World J..

[87] Petropoulos S.A., Karkanis A., Fernandes Â., Barros L., Ferreira I.C.F.R., Ntatsi G., Petrotos K., Lykas C., Khah E. (2015). Chemical Composition and Yield of Six Genotypes of Common Purslane (Portulaca oleracea L.): An Alternative Source of Omega-3 Fatty Acids. Plant Foods Hum. Nutr..

[88] Petropoulos S.A., Fernandes Â., Arampatzis D.A., Tsiropoulos N.G., Petrović J., Soković M., Barros L., Ferreira I.C.F.R. (2020). Seed oil and seed oil byproducts of common purslane (Portulaca oleracea L.): A new insight to plant-based sources rich in omega-3 fatty acids. LWT.

[89] Prasad P., Anjali P., Sreedhar R.V. (2020). Plant-based stearidonic acid as sustainable source of omega-3 fatty acid with functional outcomes on human health. Crit. Rev. Food Sci. Nutr..

[90] Rincón-Cervera M.Á., Galleguillos-Fernández R., González-Barriga V., Valenzuela R., Speisky H., Fuentes J., Valenzuela A. (2020). Fatty Acid Profile and Bioactive Compound Extraction in Purple Viper's Bugloss Seed Oil Extracted with Green Solvents. J. Am. Oil Chem. Soc..

[91] Guil-Guerrero J.L., Gómez-Mercado F., Ramos-Bueno R.P., Rincón-Cervera M.Á., Venegas-Venegas E. (2014). Restricted-Range Boraginaceae Species Constitute Potential Sources of Valuable Fatty Acids. J. Am. Oil Chem. Soc..

[92] Sreedhar R.V., Prasad P., Reddy L.P.A., Rajasekharan R., Srinivasan M. (2017). Unravelling a stearidonic acid-rich triacylglycerol biosynthetic pathway in the developing seeds of Buglossoides arvensis: A transcriptomic landscape. Sci. Rep..

[93] Guil-Guerrero J.L., Gómez-Mercado F., Ramos-Bueno R.P., González-Fernández M.J., Urrestarazu M., Jiménez-Becker S., de Bélair G. (2018). Fatty acid profiles and sn-2 fatty acid distribution of γ-linolenic acid-rich Borago species. J. Food Compos. Anal..

[94] Piskernik S., Vidrih R., Demsar L., Koron D., Rogelj M., Zontar T.P. (2018). Fatty acid profiles of seeds from different Ribes species. LWT—Food Sci. Technol..

[95] Park H.Y., Kim D.H., Saini R.K., Gopal J., Keum Y.S., Sivanesan I. (2019). Micropropagation and Quantification of Bioactive Compounds in *Mertensia maritima* (L.) Gray. Int. J. Mol. Sci..

[96] Leyland B., Leu S., Boussiba S. (2017). Are Thraustochytrids algae?. Fungal Biol..

[97] Lopes da Silva T., Moniz P., Silva C., Reis A. (2019). The Dark Side of Microalgae Biotechnology: A Heterotrophic Biorefinery Platform Directed to ω-3 Rich Lipid Production. Microorganisms.

[98] Russo G.L., Langellotti A.L., Oliviero M., Sacchi R., Masi P. (2021). Sustainable production of food grade omega-3 oil using aquatic protists: Reliability and future horizons. New Biotechnol..

[99] Patel A., Karageorgou D., Katapodis P., Sharma A., Rova U., Christakopoulos P., Matsakas L. (2021). Bioprospecting of thraustochytrids for omega-3 fatty acids: A sustainable approach to reduce dependency on animal sources. Trends Food Sci. Technol..

[100] Gray R.J. (2017). Application for the Authorization of DHA and EPA-Rich Algal oil from Schizochytrium sp..

[101] Huang T.Y., Lu W.C., Chu I.M. (2012). A fermentation strategy for producing docosahexaenoic acid in Aurantiochytrium limacinum SR21 and increasing C22:6 proportions in total fatty acid. Bioresour. Technol..

[102] FSANZ (2003). DHASCO and ARASCO Oils as Sources of Long-Chain Polyunsaturated Fatty Acids in Infant Formula.

[103] Kiy T., Luy M., Zeumer O. (2010). Production of Omega-3 Fatty Acids in Microflora of Thraustochytriales Using Modified Media. Google Patents. https://data.epo.org/gpi/EP2084290B1.

[104] Li J., Liu R., Chang G., Li X., Chang M., Liu Y., Jin Q., Wang X. (2015). A strategy for the highly efficient production of docosahexaenoic acid by Aurantiochytrium limacinum SR21 using glucose and glycerol as the mixed carbon sources. Bioresour. Technol..

[105] Patel A., Liefeldt S., Rova U., Christakopoulos P., Matsakas L. (2020). Co-production of DHA and squalene by thraustochytrid from forest biomass. Sci. Rep..

[106] Scott S.D., Armenta R.E., Berryman K.T., Norman A.W. (2011). Use of raw glycerol to produce oil rich in polyunsaturated fatty acids by a thraustochytrid. Enzym. Microb. Technol..

[107] Ma X.-N., Chen T.-P., Yang B., Liu J., Chen F. (2016). Lipid Production from Nannochloropsis. Mar. Drugs.

[108] Paliwal C., Mitra M., Bhayani K., Bharadwaj S.V.V., Ghosh T., Dubey S., Mishra S. (2017). Abiotic stresses as tools for metabolites in microalgae. Bioresour. Technol..

[109] Mitra M., Patidar S.K., Mishra S. (2015). Integrated process of two stage cultivation of Nannochloropsis sp. for nutraceutically valuable eicosapentaenoic acid along with biodiesel. Bioresour. Technol..

[110] Cui Y., Thomas-Hall S.R., Schenk P.M. (2019). Phaeodactylum tricornutum microalgae as a rich source of omega-3 oil: Progress in lipid induction techniques towards industry adoption. Food Chem..

[111] Maehre H.K., Malde M.K., Eilertsen K.-E., Elvevoll E.O. (2014). Characterization of protein, lipid and mineral contents in common Norwegian seaweeds and evaluation of their potential as food and feed. J. Sci. Food Agric..

[112] Pereira H., Barreira L., Figueiredo F., Custódio L., Vizetto-Duarte C., Polo C., Rešek E., Engelen A., Varela J. (2012). Polyunsaturated Fatty Acids of Marine Macroalgae: Potential for Nutritional and Pharmaceutical Applications. Mar. Drugs.

[113] Gosch B.J., Magnusson M., Paul N.A., De Nys R. (2012). Total lipid and fatty acid composition of seaweeds for the selection of species for oil-based biofuel and bioproducts. GCB Bioenergy.

[114] Umesha S.S., Monahar B., Naidu K.A. (2013). Microencapsulation of α-linolenic acid-rich garden cress seed oil: Physical characteristics and oxidative stability. Eur. J. Lipid Sci. Technol..

[115] Lyashenko S., González-Fernández M.J., Borisova S., Belarbi E.-H., Guil-Guerrero J.L. (2021). Mertensia (Boraginaceae) seeds are new sources of γ-linolenic acid and minor functional compounds. Food Chem..

[116] Lim D.K.Y., Garg S., Timmins M., Zhang E.S.B., Thomas-Hall S.R., Schuhmann H., Li Y., Schenk P.M. (2012). Isolation and Evaluation of Oil-Producing Microalgae from Subtropical Coastal and Brackish Waters. PLoS ONE.

[117] Qiao H., Cong C., Sun C., Li B., Wang J., Zhang L. (2016). Effect of culture conditions on growth, fatty acid composition and DHA/EPA ratio of Phaeodactylum tricornutum. Aquaculture.

[118] Patel A., Mu L., Shi Y., Rova U., Christakopoulos P., Matsakas L. (2020). Novel Biorefinery Approach Aimed at Vegetarians Reduces the Dependency on Marine Fish Stocks for Obtaining Squalene and Docosahexaenoic Acid. ACS Sustain. Chem. Eng..

[119] Schmid M., Guihéneuf F., Stengel D.B. (2014). Fatty acid contents and profiles of 16 macroalgae collected from the Irish Coast at two seasons. J. Appl. Phycol..

[120] Ruiz-Lopez N., Sayanova O., Napier J.A., Haslam R.P. (2012). Metabolic engineering of the omega-3 long chain polyunsaturated fatty acid biosynthetic pathway into transgenic plants. J. Exp. Bot..

[121] Haslam R.P., Ruiz-Lopez N., Eastmond P., Moloney M., Sayanova O., Napier J.A. (2013). The modification of plant oil composition via metabolic engineering-better nutrition by design. Plant Biotechnol. J..

[122] Petrie J.R., Shrestha P., Belide S., Kennedy Y., Lester G., Liu Q., Divi U.K., Mulder R.J., Mansour M.P., Nichols P.D. (2014). Metabolic Engineering Camelina sativa with Fish Oil-Like Levels of DHA. PLoS ONE.

[123] Usher S., Han L., Haslam R.P., Michaelson L.V., Sturtevant D., Aziz M., Chapman K.D., Sayanova O., Napier J.A. (2017). Tailoring seed oil composition in the real world: Optimising omega-3 long chain polyunsaturated fatty acid accumulation in transgenic Camelina sativa. Sci. Rep..

[124] Han L., Usher S., Sandgrind S., Hassall K., Sayanova O., Michaelson L.V., Haslam R.P., Napier J.A. (2020). High level accumulation of EPA and DHA in field-grown transgenic Camelina—A multi-territory evaluation of TAG accumulation and heterogeneity. Plant Biotechnol. J..

[125] Xie D., Jackson E.N., Zhu Q. (2015). Sustainable source of omega-3 eicosapentaenoic acid from metabolically engineered Yarrowia lipolytica: From fundamental research to commercial production. Appl. Microbiol. Biotechnol..

[126] Ganesan B., Brothersen C., McMahon D.J. (2014). Fortification of Foods with Omega-3 Polyunsaturated Fatty Acids. Crit. Rev. Food Sci. Nutr..

[127] Wang Y.Z., Fu S.G., Wang S.Y., Yang D.J., Wu Y.H.S., Chen Y.C. (2018). Effects of a natural antioxidant, polyphenol-rich rosemary (Rosmarinus officinalis L.) extract, on lipid stability of plant-derived omega-3 fatty-acid rich oil. LWT—Food Sci. Technol..

[128] Shen Y., Lu T., Liu X.-Y., Zhao M.-T., Yin F.-W., Rakariyatham K., Zhou D.-Y. (2020). Improving the oxidative stability and lengthening the shelf life of DHA algae oil with composite antioxidants. Food Chem..

[129] Nain C.W., Berdal G., Thao P.T.P., Mignolet E., Buchet M., Page M., Larondelle Y. (2021). Green Tea Extract Enhances the Oxidative Stability of DHA-Rich Oil. Antioxidants.

[130] Mikołajczak N., Sobiechowska D.A., Tańska M. (2020). Edible flowers as a new source of natural antioxidants for oxidative protection of cold-pressed oils rich in omega-3 fatty acids. Food Res. Int..

[131] Yui Y., Miyazaki S., Ma Y., Ohira M., Fiehn O., Ikegami T., McCalley D.V., Tanaka N. (2016). Distinction of synthetic dl-α-tocopherol from natural vitamin E (d-α-tocopherol) by reversed-phase liquid chromatography. Enhanced selectivity of a polymeric C18 stationary phase at low temperature and/or at high pressure. J. Chromatogr. A.

[132] Jurić S., Jurić M., Siddique M.A.B., Fathi M. (2020). Vegetable Oils Rich in Polyunsaturated Fatty Acids: Nanoencapsulation Methods and Stability Enhancement. Food Rev. Int..

[133] Kaushik P., Dowling K., Barrow C.J., Adhikari B. (2015). Microencapsulation of omega-3 fatty acids: A review of microencapsulation and characterization methods. J. Funct. Foods.

[134] Geranpour M., Assadpour E., Jafari S.M. (2020). Recent advances in the spray drying encapsulation of essential fatty acids and functional oils. Trends Food Sci. Technol..

[135] Chang C., Nickerson M.T. (2018). Encapsulation of omega 3-6-9 fatty acids-rich oils using protein-based emulsions with spray drying. J. Food Sci. Technol..

[136] Feizollahi E., Hadian Z., Honarvar Z. (2018). Food fortification with omega-3 fatty acids; microencapsulation as an addition method. Curr. Nutr. Food Sci..

[137] Bakry A.M., Abbas S., Ali B., Majeed H., Abouelwafa M.Y., Mousa A., Liang L. (2016). Microencapsulation of Oils: A Comprehensive Review of Benefits, Techniques, and Applications. Compr. Rev. Food Sci. Food Saf..

[138] Rodríguez J., Martín M.J., Ruiz M.A., Clares B. (2016). Current encapsulation strategies for bioactive oils: From alimentary to pharmaceutical perspectives. Food Res. Int..

[139] Gulotta A., Saberi A.H., Nicoli M.C., McClements D.J. (2014). Nanoemulsion-Based Delivery Systems for Polyunsaturated (ω-3) Oils: Formation Using a Spontaneous Emulsification Method. J. Agric. Food Chem..

[140] Inapurapu S.P., Ibrahim A., Kona S.R., Pawar S.C., Bodiga S., Bodiga V.L. (2020). Development and characterization of ω-3 fatty acid nanoemulsions with improved physicochemical stability and bioaccessibility. Colloids Surf. A Physicochem. Eng. Asp..

[141] Cholewski M., Tomczykowa M., Tomczyk M. (2018). A Comprehensive Review of Chemistry, Sources and Bioavailability of Omega-3 Fatty Acids. Nutrients.

[142] Schuchardt J.P., Hahn A. (2013). Bioavailability of long-chain omega-3 fatty acids. Prostaglandins Leukot. Essent. Fat. Acids.

[143] Lee-Chang K.J., Taylor M.C., Drummond G., Mulder R.J., Mansour M.P., Brock M., Nichols P.D. (2021). Docosahexaenoic Acid Is Naturally Concentrated at the sn-2 Position in Triacylglycerols of the Australian Thraustochytrid Aurantiochytrium sp. Strain TC 20. Mar. Drugs.

[144] Jin J., Jin Q., Wang X., Akoh C.C. (2020). High Sn-2 Docosahexaenoic Acid Lipids for Brain Benefits, and Their Enzymatic Syntheses: A Review. Engineering.

[145] Alfieri A., Imperlini E., Nigro E., Vitucci D., Orrù S., Daniele A., Buono P., Mancini A. (2018). Effects of Plant Oil Interesterified Triacylglycerols on Lipemia and Human Health. Int. J. Mol. Sci..

[146] Kuhnt K., Fuhrmann C., Köhler M., Kiehntopf M., Jahreis G. (2014). Dietary Echium Oil Increases Long-Chain n–3 PUFAs, Including Docosapentaenoic Acid, in Blood Fractions and Alters Biochemical Markers for Cardiovascular Disease Independently of Age, Sex, and Metabolic Syndrome. J. Nutr..

[147] Dittrich M., Jahreis G., Bothor K., Drechsel C., Kiehntopf M., Blüher M., Dawczynski C. (2015). Benefits of foods supplemented with vegetable oils rich in α-linolenic, stearidonic or docosahexaenoic acid in hypertriglyceridemic subjects: A double-blind, randomized, controlled trail. Eur. J. Nutr..

[148] Maki K.C., Yurko-Mauro K., Dicklin M.R., Schild A.L., Geohas J.G. (2014). A new, microalgal DHA- and EPA-containing oil lowers triacylglycerols in adults with mild-to-moderate hypertriglyceridemia. Prostaglandins Leukot. Essent. Fat. Acids.

[149] Ryan L., Symington A.M. (2015). Algal-oil supplements are a viable alternative to fish-oil supplements in terms of docosahexaenoic acid (22:6n-3; DHA). J. Funct. Foods.

[150] Khandelwal S., Kondal D., Chaudhry M., Patil K., Swamy M.K., Metgud D., Jogalekar S., Kamate M., Divan G., Gupta R. (2020). Effect of Maternal Docosahexaenoic Acid (DHA) Supplementation on Offspring Neurodevelopment at 12 Months in India: A Randomized Controlled Trial. Nutrients.

[151] Dewell A., Marvasti F.F., Harris W.S., Tsao P., Gardner C.D. (2011). Low- and High-Dose Plant and Marine (n-3) Fatty Acids Do Not Affect Plasma Inflammatory Markers in Adults with Metabolic Syndrome. J. Nutr..

[152] Ghobadi S., Hassanzadeh-Rostami Z., Mohammadian F., Zare M., Faghih S. (2019). Effects of Canola Oil Consumption on Lipid Profile: A Systematic Review and Meta-Analysis of Randomized Controlled Clinical Trials. J. Am. Coll. Nutr..

[153] Günç Ergönül P., Aksoylu Özbek Z., Ramadan M.F. (2020). Chapter 21—Cold pressed camelina (*Camelina sativa* L.) seed oil. Cold Pressed Oils.

[154] Shetty U.S., Akhilender N.K. (2017). Garden cress (*Lepidium sativum* L.) Seed Oil: Alternative Source for ALA. FASEB J..

[155] Copetta A., Bazzicalupo M., Cassetti A., Marchioni I., Mascarello C., Cornara L., Pistelli L., Ruffoni B. (2021). Plant Production and Leaf Anatomy of *Mertensia maritima* (L.) Gray: Comparison of In Vitro Culture Methods to Improve Acclimatization. Horticulturae.

